# Bipolar sealer not superior to standard electrocautery in primary total hip arthroplasty: a meta-analysis

**DOI:** 10.1186/s13018-014-0092-5

**Published:** 2014-10-10

**Authors:** Yang Yang, Li-chao Zhang, Fei Xu, Jia Li, Yong-ming Lv

**Affiliations:** Orthopedic Department, The Affiliated Hospital of Chengde Medical College, Chengde, 067700 People’s Republic of China

**Keywords:** Hip arthroplasty, Bipolar sealer, Hemostasis, Meta-analysis

## Abstract

**Introduction:**

To assess whether bipolar sealer has advantages over standard electrocautery in primary total hip arthroplasty (THA).

**Methods:**

All studies published through November 2013 were systematically searched in PubMed, Embase, ScienceDirect, The Cochrane Library, and other databases. Relevant journals or conference proceedings were searched manually. Only randomized controlled trials were included. Two independent reviewers identified and assessed the literature. Mean difference in blood loss and risk ratios of transfusion rates and of complication rates in the bipolar sealer group versus the standard electrocautery group were calculated. The meta-analysis was conducted using RevMan 5.1 software.

**Results:**

Five studies were included, with a total sample size of 559 patients. The use of bipolar sealer did not significantly reduce intraoperative blood loss, hemoglobin drop, hospital stay, and operative time. There were no significant differences in need for transfusion and the incidence of infection between the study groups.

**Conclusion:**

The available evidence suggests that the use of bipolar sealer was not superior to standard electrocautery in patients undergoing primary THA. The use of bipolar sealer is not recommended in primary THA.

## Introduction

Total hip arthroplasty (THA) has become a common treatment for hip disorders such as severe osteoarthritis, rheumatoid arthritis, femoral head necrosis, and femoral neck fractures. Because of extensive soft tissue dissection, long operative times, and operation on the bone, patients undergoing primary THA are particularly prone to large blood loss, from 1,000 to 2,000 ml [1-4].

Several blood-preservation techniques are in regular clinical use: hemostatic agents, erythropoietic agents, minimally invasive surgery, intraoperative and postoperative salvage of blood with reinfusion, hypotensive or epidural anesthesia, and preoperative autologous blood donation [5-7]. Generally speaking, patients need blood transfusion because of intra- and/or postoperative blood loss. Transfused patients are exposed to risks such as adverse immunological reactions, disease transmission, intravascular hemolysis, transfusion-induced coagulopathy, renal impairment or failure, and even increased mortality [8-10]. Effective hemostasis in THA results in lower risk of blood transfusion and, therefore, faster postoperative recovery and lower medical costs.

Standard electrocautery is commonly adopted to achieve intraoperative hemostasis. However, standard electrocautery has been reported to cause severe burns and severe tissue necrosis in patients, and operating room fires; moreover, viruses and carcinogens have been detected in the smoke generated by the device during surgery [11-14]. In addition, investigators have also noted that skin incision healing is quite slow, because of which, they argued, application of electrocautery should be limited to reduce the postoperative complications [15]. Compared with conventional electrocautery, bipolar sealer may achieve hemostasis at 100°C or lower temperatures. Bipolar sealer avoids charring or burning tissue, does not produce smoke, and may transport radiofrequency energy to saline for hemostatic sealing and coagulation of soft tissue [16]. Consequently, it is favored in hepatic transplantations, cirrhotic liver resections, cholecystectomies, and oncological surgery.

To our knowledge, numerous prospective randomized controlled trials (RCTs) have focused on the use of bipolar sealer in primary THA. However, the results are not consistent. Studies have been criticized for poor design and small sample size with consequently low power. Therefore, we conducted a meta-analysis, pooling the data from RCTs to provide an evidence-based judgment regarding the use of bipolar sealer in patients undergoing primary THA.

## Methods

### Search strategy

**Figure 1:**
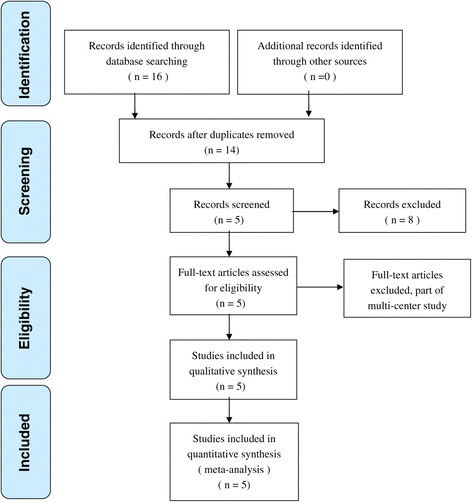
**Flowchart of the study selection.**

We conducted a meta-analysis to identify academic articles from electronic databases, including MEDLINE (1966 to November 2013), Embase (1980 to November 2013), and The Cochrane Central Register of Controlled Trials. There were no language restrictions. The search strategy is presented in Figure 1. It included only studies conducted on human subjects. In addition, using the Google search engine, the same search terms were manually searched to find any further relevant studies that may have been missed in the database search. We used the following key words: “hip replacement OR arthroplasty” and “bipolar sealer” in combination with Boolean operators AND or OR.

### Selection criteria and quality assessment

We included all published RCTs and quasi-RCTs (trials using a method of allocating participants to a treatment that is not strictly random, e.g., by date of birth, hospital record number, alternation) comparing the bipolar sealer with standard electrocautery in patients undergoing THA. Exclusion criteria comprised the following (by implication): trials with retrospective design, those without randomization of patients into two relevant groups, together with studies focusing on an orthopedic. The methodological quality of the included studies was assessed by the review authors using a modification of the generic evaluation tool used by the Cochrane Bone, Joint and Muscle Trauma Group. To provide a qualification of bias risk, quality criteria included (i) details of randomization method, (ii) allocation concealment, (iii) blinding of participants and personnel, (iv) blind outcome assessment, (v) incomplete outcome data, (vi) selective outcome reporting, and (vii) other sources of bias.

### Data extraction

For each eligible study, two of the authors independently extracted all the relevant data. Disagreement was resolved by discussion with the third reviewer. Whenever necessary, we contacted the authors of the studies for missing data or further information. The following data were extracted: (1) demographic data of participants; (2) indication for THA; (3) wound infection (superficial or deep), hematoma, wound dehiscence, limb swelling, bleeding from the wound, reoperation because of a wound-healing complication; (4) postoperative blood transfusion, decrease in hemoglobin or hematocrit, thromboembolic complications, patient discomfort, costs; (5) functional outcomes such as time to regain mobility; and (6) any other outcomes as mentioned in individual studies were considered for inclusion. In studies in which data were incomplete or unclear, attempts were made to contact investigators for clarification.

### Data analysis and statistical methods

The meta-analysis was undertaken using RevMan 5.1 for Windows (The Cochrane Collaboration, Oxford, United Kingdom). We assessed statistical heterogeneity for each study with the use of a standard chi-square test (for heterogeneity, a level of *P* <0.1 was considered significant) and the *I*^2^ statistic. An *I*^2^ statistic value of 50% was considered to indicate substantial heterogeneity. The origins of heterogeneity, if present, were analyzed according to differences in methodological quality, characteristics of participants and intervention. When the data allowed, the authors of this paper performed subgroup analysis of the trials. If comparing trials showed heterogeneity, pooled data were meta-analyzed using a random-effects model. Otherwise, a fixed-effects model was used for the analysis. Relative risks (or risk differences) and 95% confidence intervals (CIs) were calculated for dichotomous outcomes and mean differences (MDs) and 95% CIs for continuous outcomes.

## Results

### Search results

**Figure 2:**
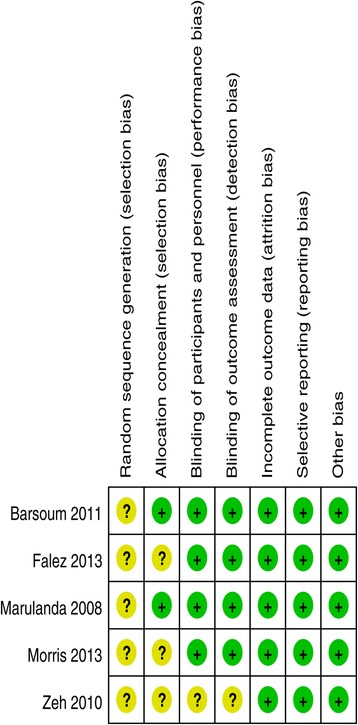
**Methodological quality of the included studies.** This risk-of-bias tool incorporates assessment of randomization (sequence generation and allocation concealment), blinding (participants, personnel and outcome assessors), completeness of outcome data, selection of outcomes reported, and other sources of bias. The items were scored with “yes”, “no”, or “unsure”.

**Figure 3:**
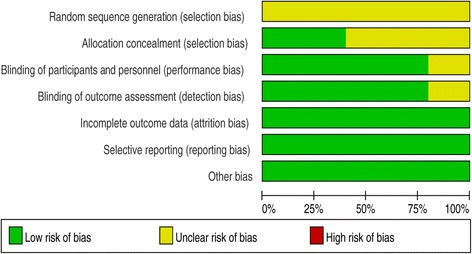
**Risk of bias.** Each risk-of-bias item presented as percentages across all included studies, which indicated the proportion of different levels of risk of bias for each item.

We identified a total of 16 citations as potentially relevant. By screening the title, and reading the abstract and the entire article, we found that five RCTs enrolling a total of 559 hips at final follow-up were eligible for data extraction and meta-analysis [17-21] (Figure 1). The sample size for each study ranged from 50 to 200. Studies were relatively well designed, and the quality assessment score was high. However, the relevant RCTs had a number of methodological weaknesses (Figures 2 and 3).

None of the RCTs provided randomization methods. Two studies reported allocation concealment using sealed envelopes [17,19]. Four studies attempted to blind the subjects and assessors to group allocation [17-20]. All studies reported final outcomes for a minimum of 85% of their randomized patients.

Inclusion and exclusion criteria were reported for all studies. The patients’ characteristics were comparable within each study group and are presented in Table 1. Two studies reported a transfusion trigger, which was related to a fall in either hemoglobin levels or clinical symptoms [17,20]. The blood transfusion protocol was not mentioned in three studies. For prophylaxis against deep vein thrombosis, three studies used low-molecular-weight heparin (LMWH) [17,18,21], one study used aspirin [20], and one study did not provide information. Two studies mentioned anesthesia: one used spinal anesthesia [18], and the other used general and spinal anesthesia [21].

**Table 1:** **Characteristics of the included studies**

	**Cases (BP/C)**	**Mean age (BP/C)**	**Male patients (BP/C)**	**Prosthesis**	**DVT prophylaxis**	**Length of follow-up**
Barsoum et al. (2011) [17]	71/69	55.4/55.7	36/38	N/A	Enoxaparin	12 weeks
Marulanda et al. (2008) [19]	25/25	57/56	13/14	N/A	N/A	N/A
Morris et al. (2013) [20]	100/100	63.5/61.3	48/48	N/A	Aspirin	6 weeks
Zeh et al. (2010) [21]	55/50	63.7/68.3	15/20	Cementless hybrid	Enoxaparin	N/A
Falez et al. (2013) [18]	26/38	N/A	N/A	Cementless	Enoxaparin	N/A

*N/A* not available, *DVT* deep vein thrombosis.

Different studies used different surgical approaches. Zeh et al. reported that a minimally invasive modified Watson-Jones approach and a standard Bauer approach were performed. Morris et al. used an anterior supine intermuscular approach. Falez et al. used a direct lateral approach in the lateral position [18]. Marulanda et al. used the anterolateral approach [19]. Barsoum et al. used a standard posterior approach and a modified direct lateral approach [17].

### Meta-analysis results

#### Hemoglobin drop

**Figure 4:**
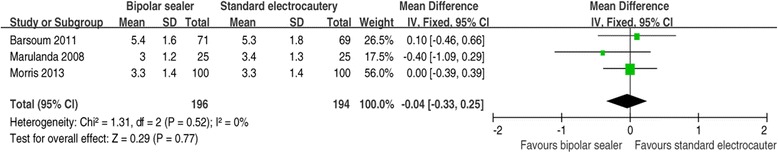
**Forest plot showing the effect of bipolar sealer on hemoglobin drop.**

We obtained usable data on hemoglobin drop from three trials including 390 hips [17,19,20]. As depicted in Figure 4, there was no significant heterogeneity (*χ*^2^ = 1.31, df =2, *I*^2^ = 0%, *P* =0.52). Using a fixed-effects model, the pooled results indicated that there was no significant difference between the groups in terms of hemoglobin drop (MD = −0.04, 95% CI: −0.33 to 0.25, *P* =0.77).

#### Hospital stay

**Figure 5:**
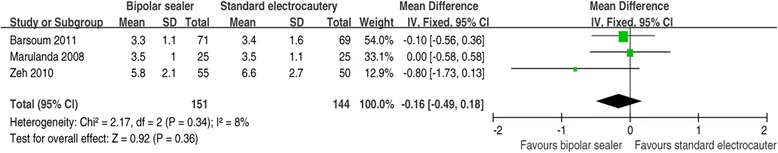
**Forest plot showing the effect of bipolar sealer on hospital stay.**

Hospital stay was mentioned in three trials [17,19,21]. The pooled results showed no significant heterogeneity (*χ*^2^ = 2.17, df =2, *I*^2^ = 8%, *P* =0.34; Figure 5); thus, a fixed-effects model was used. Meta-analysis showed no significant difference between the groups in terms of hospital stay (MD = −0.16, 95% CI: −0.49 to 0.18, *P* =0.36).

#### Infection

**Figure 6:**
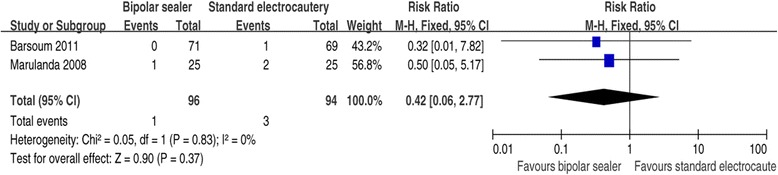
**Forest plot showing the effect of bipolar sealer on intraoperative blood loss.**

The incidence of infection was reported in two studies [17,19]. The pooled results indicated that the incidence of infection was 1.04% of hips (1/96) in the bipolar sealer group, compared with 3.19% (3/94) in the conventional group. This difference was significant (RR =0.42, 95% CI: 0.06 to 2.77, *P* =0.37; Figure 6). A fixed-effects model was used because no statistical heterogeneity was found between the studies (*χ*^2^ = 0.05, df =1, *I*^2^ = 0%, *P* =0.83).

#### Intraoperative blood loss

**Figure 7:**
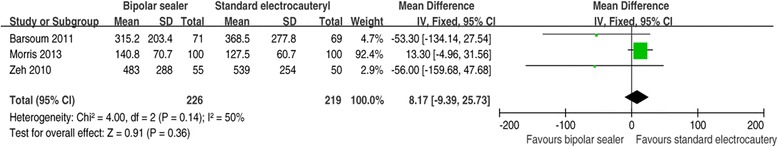
**Forest plot showing the effect of bipolar sealer on infection.**

Intraoperative blood loss was documented in three studies [17,20,21]. The difference between the groups was not significant (MD =8.17, 95% CI: −9.39 to 25.73, *P* =0.36; Figure 7). A fixed-effects model was used because no statistical heterogeneity was found between the studies (*χ*^2^ = 4.00, df =2, *I*^2^ = 50%, *P* =0.14).

#### Need for transfusion

**Figure 8:**
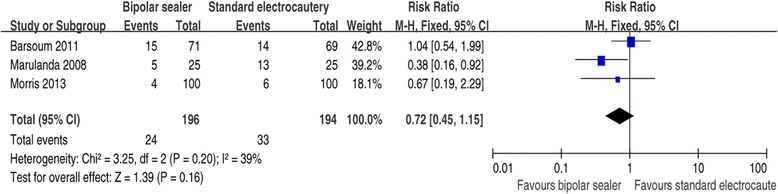
**Forest plot showing the effect of bipolar sealer on operative time.**

Need for transfusion was reported in three trials [17,19,20]. The pooled results showed a significant difference between the groups (RR =0.72, 95% CI: 0.45 to 1.15, *P* =0.16). There was no significant heterogeneity (*χ*^2^ = 3.25, df =2, *I*^2^ = 39%, *P* =0.20; Figure 8). A fixed-effects model was used.

#### Operating time

**Figure 9:**
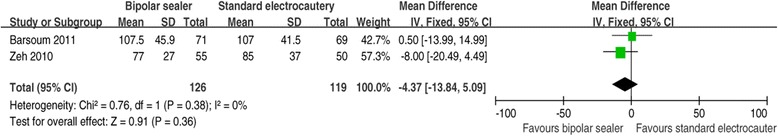
**Forest plot showing the effect of bipolar sealer on need for transfusion.**

Operative time was reported in two trials [17,21]. One study mentioned that the use of bipolar sealer resulted in a longer operative time (6 min), but did not give means and standard deviations [19]. The pooled results of the other trials showed that the use of bipolar sealer did not extend operative time (MD = −4.37 min, 95% CI: −13.84 to 5.09, *P* =0.36) compared to that of conventional surgery (Figure 9). A fixed-effects model was used because statistical heterogeneity was found between the studies (*χ*^2^ = 0.76, df =1, *I*^2^ = 0%, *P* =0.38).

#### Other outcomes

Several other outcome measures were identified, but insufficient data were provided for meta-analysis. For instance, Barsoum et al. found that there were no significant differences in the requirement for the number of units transfused, Harris hip score, pain score, and Short Form-12 score [17]. Marulanda et al. reported fewer units transfused, less intraoperative blood loss, less drainage and less total blood loss in the bipolar sealer group, and no statistical differences in Harris hip score between the two groups [19].

## Discussion

The most important finding of the present meta-analysis was that bipolar sealer in primary THA did not reduce hemoglobin drop, intraoperative blood loss, need for transfusion, and hospital stay. Furthermore, no significant differences were found in operating time and infection. The effectiveness of bipolar sealer in primary THA is questionable.

Five RCTs satisfied the defined eligibility criteria for this meta-analysis. The overall methodological quality of the included studies was relatively high. Although all of the studies reported randomization, none described the method of randomization used. Therefore, the available information left us unsure whether the right randomization methods had been used. Two studies reported allocation concealment. Four studies attempted to blind the subjects and assessors to group allocation, which reduced expectation bias and the potential for type II statistical error in their clinical outcomes. All included studies had consistent baseline data, and intention to treat analysis was performed for withdrawals and dropouts. These methodological strengths and weaknesses should be considered when interpreting the findings of the present meta-analysis.

Primary total hip replacement is complicated by perioperative blood loss ranging in amount from 1,000 to 2,000 ml. Blood transfusion was needed to correct anemia in 3% to 50% of patients [22]. This meta-analysis showed that bipolar sealer did not reduce intraoperative blood loss (MD =8.17, 95% CI: −9.39 to 25.73, *P* =0.36) and hemoglobin drop (MD = −0.04, 95% CI: −0.33 to 0.25, *P* =0.77) in primary THA compared with standard electrocautery. Most of the included studies were consistent with this result. Marulanda et al., contesting other authors, demonstrated lower intraoperative blood loss and hemoglobin drop with the use of a bipolar sealer; however, they did not provide standard deviation of intraoperative blood loss for meta-analysis.

In the present meta-analysis, we found that patients in the standard electrocautery group were not significantly more likely to receive an allogeneic blood transfusion than those in the bipolar sealer group (RR =0.72, 95% CI: 0.45 to 1.15, *P* =0.16). Two of the included studies agreed. This result contests that of Marulanda et al., who reported that more patients in the control group needed transfusion than did patients in the bipolar sealer group (52% vs. 20%). Their finding may be due to greater total blood loss (1,067 vs. 662 ml) and hemoglobin drop (3.4 g/dl vs. 3.0 g/dl) in the controls after THA.

Infection is relatively rare after THA but can be devastating in terms of morbidity and cost [23]. In theory, bipolar sealer avoids the disadvantages of standard electrocautery. This meta-analysis found no significant difference in the incidence of infection, which was 1.04% with bipolar sealer and 3.19% in controls; the overall infection rate was 2.1%. The reported incidence of infection after THA ranges from 0.6% to 3% [24]. Because infection may also occur later, assessment after a longer follow-up period may be required.

Three studies mentioned the mean and standard deviation of the length of hospital stay, but the pooled data in this meta-analysis found no significant differences, which was consistent with their results. Two studies reported the operating time. Our results also suggest that bipolar sealer did not significantly decrease operative time.

There are several potential limitations of our meta-analysis. (1) Only five reports were included, and their sample sizes were small, which may have affected our conclusions. (2) The follow-up of patients in some of the trials was unclear. Many patients were followed up in the short term. This may have resulted in underreporting of, for example, infection. (3) There were insufficient data to support analyses of functional outcome scores, cost, drainage, postoperative swelling, and pain relief as had originally been planned. However, this is the first systematic review to evaluate the administration of bipolar sealer during THA by only including studies that have appropriate control and study groups. All of the included studies were high-quality RCTs with good homogeneity.

## Conclusion

In summary, the using of bipolar sealer was not superior to standard electrocautery in patients undergoing primary THA. The use of bipolar sealer is not recommended in primary THA. Because of the limited quality of the evidence currently available, more high quality RCTs with better experimental design, larger patient samples and longer follow-up are required.
